# Rheumatoid nodules in the thyroid bed following total thyroidectomy: a case report

**DOI:** 10.1186/1752-1947-7-247

**Published:** 2013-10-29

**Authors:** Amit Bhargava, Poornima Upendra Hegde, Sameera Tallapureddy, Sarah Varghese, Faripour A Forouhar, Beatriz E Tendler

**Affiliations:** 1 Division of Endocrinology and Metabolism, University of Connecticut Health Center, 263 Farmington Avenue, Farmington, CT 06030, USA; 2 Division of Pathology and Laboratory Medicine, University of Connecticut Health Center, 263 Farmington Avenue, Farmington, CT 06030, USA

**Keywords:** Rheumatoid nodule, Thyroid bed, Rheumatoid arthritis

## Abstract

**Introduction:**

Rheumatoid nodules occur in 30 percent of patients with active rheumatoid arthritis. Common sites include the buttocks or the extensor surface of the forearm, with one group documenting their presence in the thyrohyoid membrane. To the best of our knowledge, rheumatoid nodules have not been described in the thyroid bed.

**Case presentation:**

We present the case of a 46-year-old Caucasian woman with active rheumatoid arthritis and Hashimoto's thyroiditis who presented with compressive neck symptoms. An ultrasound scan revealed that both lobes of her thyroid were enlarged. The right lobe measured 7.9×3.4×3.3cm and the left 8.3×3.3×3.1cm. A solitary 1.0×0.6×0.8cm nodule was seen in the right lower lobe. Her thyroid-stimulating hormone level was 4.22uU/mL (0.34 to 5.60). A total thyroidectomy was performed due to her symptoms and the possible growth of a nodule when on levothyroxine. A postoperative ultrasound scan showed no remaining thyroid tissue. The pathology revealed several small neoplasms ranging from a well-encapsulated adenoma to highly atypical follicular and papillary Hurthle cell lesions in the setting of Hashimoto’s thyroiditis. Low-dose radioactive iodine (33.4mCi) was given. Four months later, our patient complained of a feeling of fullness in her neck. A solid nodule of mixed echogenicity (5.6×3.3×2.3cm) was seen in the right level VI of the neck, and solid tissue of mixed echogenicity (2.9×2.3×1.7cm) on the left. Following repeat surgery, the pathology from the right specimen showed Hashimoto’s thyroiditis. The left specimen had areas of granuloma formation with fibrinoid necrosis and palisading histiocytes, consistent with the histology of rheumatoid nodules. No evidence of malignancy was seen. The patient continues to do well and remains disease-free.

**Conclusions:**

Rheumatoid nodules have not been reported in the thyroid bed. Their pathogenesis is not clear. Postoperative release of tumor necrosis factor alpha and local vascular damage may have triggered the nodule formation in this case. Rheumatoid nodules must be kept in the differential diagnosis of an enlarging thyroid in the setting of active rheumatoid arthritis. A fine-needle aspiration biopsy may show granuloma formation and be the most cost-effective initial diagnostic step, especially if there is a concern for malignancy. Early identification of these nodules will help decrease morbidity from unnecessary interventions and result in treatment that is both timely and appropriate.

## Introduction

Thyroid nodules are extremely common, with autopsy studies confirming their presence in 50 percent of individuals. Not all nodules are clinically palpable, with an increasing number being found incidentally on imaging [1]. These nodules may range from that of a simple cyst, to a nonfunctioning adenoma, to a thyroid neoplasm [2]. Rheumatoid nodules are subcutaneous nodules >5mm in diameter, that occur in approximately 30 percent of patients with rheumatoid arthritis (RA). Common sites include areas of pressure or repetitive irritation such as the buttocks, sacral prominences, fingers or the extensor surfaces of the forearm [3]. Case reports also document unusual sites such as the gallbladder and the upper eyelid, with one group reporting the presence of a thyroid nodule in the thyrohyoid membrane [4]. To the best of our knowledge, we report the first case of rheumatoid nodules developing in the thyroid bed of a patient following a total thyroidectomy, and discuss the cytologic and histopathologic features of a rheumatoid nodule.

## Case presentation

A 46-year-old Caucasian woman, a nonsmoker, was evaluated in our office for an enlarged thyroid gland. Her complaints included dysphagia and a sensation of airway compression when supine. Her past medical history was significant for active RA, pulmonary nodules, Hashimoto’s thyroiditis and a radical right nephrectomy due to renal cell carcinoma. Notable outpatient medications included hydroxychloroquine 200mg twice a day orally and levothyroxine 75mcg once a day orally. Her family history was positive for a goiter in her paternal grandmother. On examination, her thyroid gland was visibly enlarged and facial plethora was noted. Her initial thyroid-stimulating hormone (TSH) level was 4.22uU/mL (0.34 to 5.60), with a free thyroxine (T4) of 0.96ng/dL and free triiodothyronine (T3) of 3.5pg/mL (2.5 to 4.2). Her calcitonin level was 7.1pg/mL (0.0 to 4.6). Her microsomal antibody levels were >1000IU/mL (0.0 to 3.9). A thyroid ultrasound scan showed an enlarged gland (the right lobe measured 7.9×3.4×3.3cm, the left lobe measured 8.3×3.3×3.1cm, and the isthmus measured 2.1cm). A solitary 1.0×0.6×0.8cm nodule was seen in the right lower lobe. A fine-needle aspiration biopsy (FNAB) of this nodule revealed follicular cells, abundant colloid and lymphocytes, consistent with a nodular goiter in the setting of lymphocytic thyroiditis. Due to a concern of worsening tracheal compression and a possibly enlarging nodule on thyroid replacement therapy, a total thyroidectomy was performed (her thyroid gland weighed 79g).

**Figure 1:**
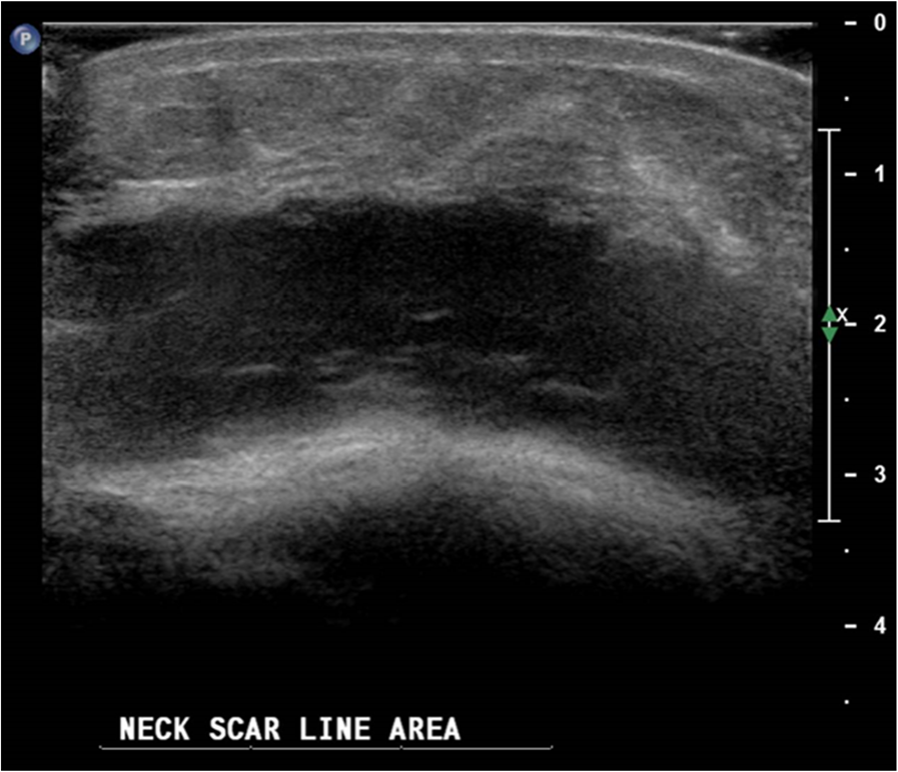
Postoperative (transverse) view of the postsurgical thyroid bed showing a seroma.

**Figure 2:**
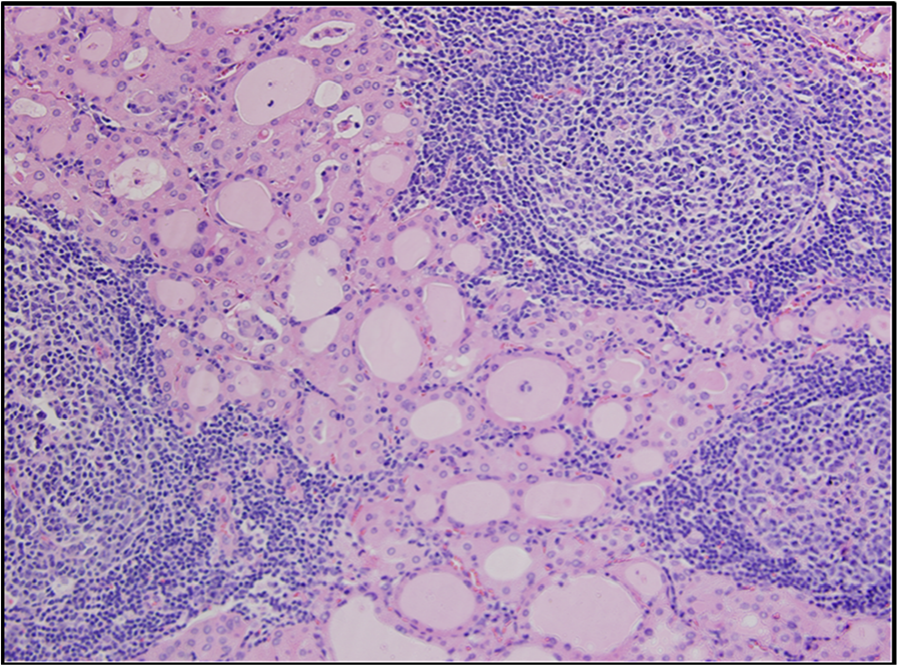
Florid Hashimoto’s thyroiditis: reactive lymphoid tissue with germinal center (hematoxylin and eosin stain, 200× magnification).

A repeat thyroid ultrasound scan showed no remaining thyroid tissue and a 5.8cm×1.4cm postoperative seroma in the thyroid bed (Figure 1). This was present in the midline of the neck, with medial and lateral extension. Postoperatively, her TSH level was 6.77uU/mL and her thyroglobulin level was 0.6ng/mL (1.8 to 68.0). However, this was difficult to interpret as a marker of residual thyroid tissue, due to the presence of thyroglobulin antibodies- 98.0IU/mL (0.0 to 14.4). Final pathology confirmed the presence of Hashimoto’s thyroiditis (Figure 2) and a 2mm papillary Hurthle cell lesion. Unequivocal evidence of carcinoma was not identified. Two reactive lymph nodes were noted at the right upper pole of her thyroid. In view of the papillary Hurthle cell lesion, our patient was treated with suppressive doses of levothyroxine (TSH was kept in the low to normal range) and low-dose radioactive iodine (RAI) (33.4mCi).

**Figure 3:**
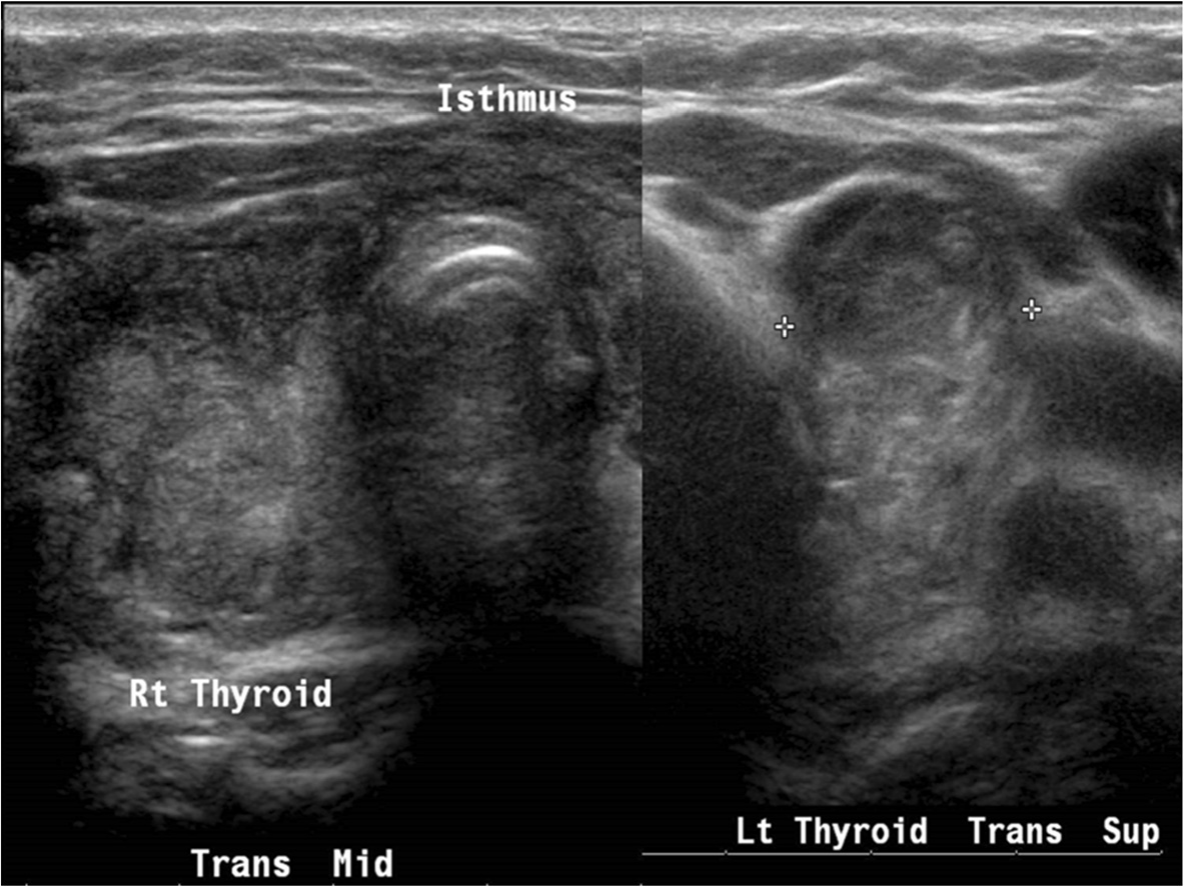
Transverse view of the thyroid bed showing a regrowth of nodular-appearing tissue (markers).

**Figure 4:**
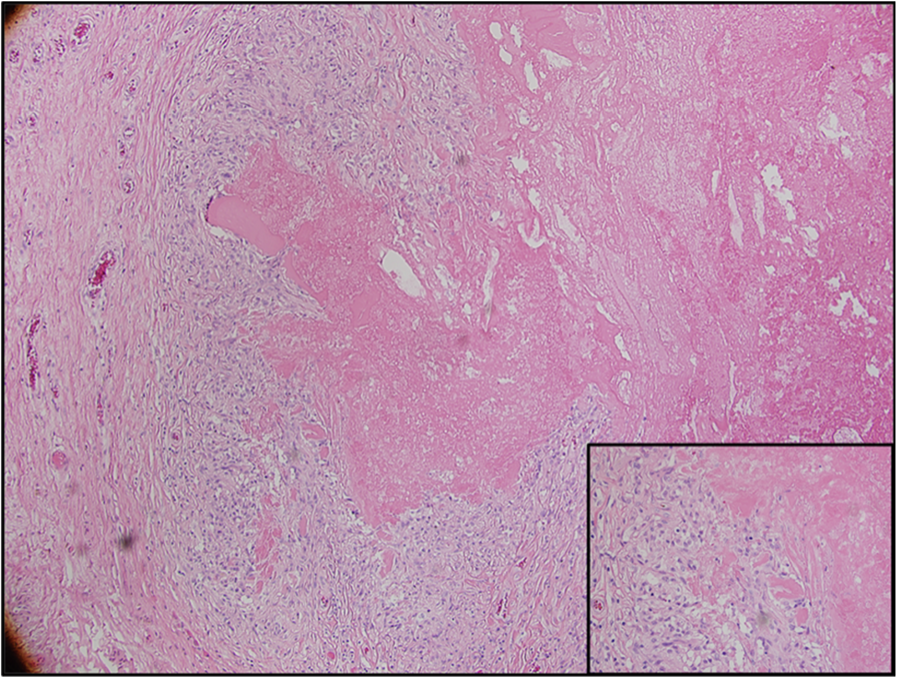
**Necrobiotic granuloma with central fibrinoid necrosis and peripheral palisaded histiocytes and occasional foreign-body-type giant cells (hematoxylin and eosin stain, 100× magnification).** Inset: Interface between necrosis and palisaded histiocytes (hematoxylin and eosin stain, 400× magnification).

Four months later, the patient again complained of fullness in her neck and a mass was felt on physical examination. A large solid nodule of mixed echogenicity, measuring 5.6×3.3×2.3cm, was seen on ultrasound scan in the right anterior compartment of her neck. In the left anterior compartment, solid tissue of mixed echogenicity was present, measuring 2.9×2.3×1.7cm. Some 4mm of tissue was also identified in the plane of the original isthmus. Due to a concern for malignancy given the rapid growth of tissue, a FNAB was not done and surgery was scheduled. Repeat surgery yielded an 11g aggregate of multiple soft, tan, irregular tissues from the right and a 1g aggregate of multiple soft, tan, irregular tissues from the left. Pathology from the right-sided tissue revealed findings consistent with Hashimoto's thyroiditis, as well as small Hurthle cell nodules (Figure 3). The left-sided tissue showed necrobiotic granulomas with central fibrinoid necrobiosis and peripheral palisaded histiocytes, consistent with rheumatoid nodules (Figure 4). Her postoperative TSH level was 5.36uU/mL (0.34 to5.60), with a thyroglobulin level of 1.0ng/mL (1.3 to 31.8). Again, this was difficult to interpret in the setting of thyroglobulin antibodies- 58.8IU/mL (0.0 to 4.4). A repeat ultrasound scan nine months later found no evidence of residual thyroid tissue in her neck. Based on the diagnosis of rheumatoid nodules within the thyroid bed, no further RAI was given. The patient continues to do well and remains disease-free.

## Discussion

Thyroid nodules are common in the general population, with an increasing number being detected through sensitive imaging techniques [1]. Total thyroidectomy has become the preferred treatment modality for bilateral benign thyroid disease and malignant thyroid disease. However, microscopic remnants of thyroid tissue will inevitably remain and can potentially cause tissue regrowth [5]. A retrospective cohort study by Marchesi also indicates that there is a risk for recurrence post thyroidectomy [6].

RA is characterized by a chronic low-grade inflammatory process that presents with both cutaneous and organ-specific extra-articular manifestations. Rheumatoid nodules occur in approximately 30 percent of patients with RA [7]. However, their presence within the thyroid bed is very rare. Common sites include the extensor surface of the forearms, over pressure points, and in the olecranon bursa. Internal organ involvement has been reported in the lungs, gallbladder and heart valves, with one case report documenting the involvement of the thyrohyoid membrane [8].

Histologically, a rheumatoid nodule consists of three zones: a central necrotic area containing cellular debris, fibrin and collagen; an intermediate area comprising of palisaded macrophages; and a surrounding area of perivascular infiltration with lymphocytes, histiocytes and plasma cells [3]. The pathogenesis behind this is not clearly defined. Trauma resulting in vascular injury and endothelial damage is thought to cause the local pooling of immunoglobulin M (IgM)-rheumatoid factor (RF) complexes on the walls of small vessels. This causes activation of macrophages and monocytes, leading to the production of interleukins and angiogenic factors such as tumor necrosis factor alpha (TNF-α). Other mediators such as the granulocyte-macrophage colony-stimulating factor, collagenase and fibronectin cause necrosis of the surrounding connective tissue matrix and contribute to the development of a palisading granuloma [7]. Nodules also contain elevated levels of interleukin (IL)-1β [8].

We feel that our case is rare as our patient developed rheumatoid nodules in the thyroid bed following a near-total thyroidectomy, along with the regrowth of thyroid tissue, in the setting of active RA. Postoperatively, the acute phase of the cytokine-mediated inflammatory response involves the release of TNF-α, IL-1 and IL-6 [9]. The release of TNF-α and local vascular damage to small blood vessels could have triggered nodule formation in this patient. Another possibility for granuloma formation in this patient would be a suture granuloma. Suture granulomas are benign inflammatory lesions that usually develop slowly, and are characterized by a histiocytic reaction to the foreign body [10]. Given the time course, pathological findings and lack of suture material in the nodule, we do not believe this was the case in our patient.

Furthermore, a possible genetic association between autoimmune thyroid disease (AITD) and RA can be postulated. Fine mapping of the AITD locus, 10q, demonstrated replicated association of the AITD phenotype (Graves’ disease and Hashimoto’s thyroiditis) and the single nucleotide polymorphism (SNP) rs6479778. In Japanese studies, this SNP, located within the *ARID5B* gene, has been associated with rheumatoid arthritis and Graves’ disease [11]. However, whether this association translates to an increase in rheumatoid nodule formation in patients with AITD is not clear. Repeat histology did not reveal the presence of any thyroid neoplasm.

Lesions such as these may pose a diagnostic challenge in patients presenting with nodules in the postsurgical thyroid bed, especially in the setting of suspected thyroid cancer. Fine-needle aspiration of such nodules might be helpful, although it was not performed in our case. Since fine-needle aspirations of rheumatoid nodules are not common, the cytologic features of such nodules may not be as well characterized as the histologic features. The age and size of the nodule affects the composition of the aspirated material. The sample should contain numerous macrophages, some with a foamy cytoplasm and others with a dense and granular cytoplasm, arranged around central cores of metachromatic necrotic material. Epithelioid histiocytes, spindle mesenchymal cells and multinucleated giant cells are also present in this central core. Well-formed granulomas are less commonly seen. Occasionally, atypia of spindle cells with hyperchromatic nuclei and large nucleoli may be seen. This can erroneously raise a suspicion of neoplasia, especially in the apt clinical setting [12].

## Conclusions

We suggest that rheumatoid nodules must be kept in the differential diagnosis of patients who present with a rapid enlargement of the thyroid gland in the setting of active RA. This is especially relevant if there is a triggering factor such as postoperative small vessel trauma. A fine-needle aspiration biopsy of the nodule may show granuloma formation, and seems to be the most appropriate and cost-effective initial next step in management. Certain cytological features can help distinguish this from neoplasia, which is often the concern with the rapid reoccurrence of tissue in the thyroid bed. Early identification of these nodules will help decrease morbidity from unnecessary interventions and result in treatment that is both timely and appropriate.

### Patient’s perspective

I am writing this patient perspective to provide assistance to the case report written about my medical experience. In October, I went to my doctor due to intense stomach pain and feeling unwell. She ordered tests, which found I had kidney cancer. I sought medical care at Hartford Hospital from a urologist, who conducted additional testing. Results from the testing indicated that I also had a problem with my thyroid. The urologist referred me to a doctor he highly regarded at the University of Connecticut, Dr. Tendler, to see me for an endocrine medical review. To say the least, my life was turned upside-down and I was overwhelmed with having two serious medical issues going on at the same time. I sought a second opinion at the Dana-Farber Cancer Institute and was assured. I was receiving excellent care by my doctor(s) in Connecticut. I had my kidney removed in November 2007 and the thyroid removed in January 2008. A few months after my thyroid was removed, I started to have neck sensations and something did not feel right. I went to see my endocrinologist and a nodule was found. It was determined that it needed to be removed based on my medical situation. I consider myself to be very lucky to have such great care coordinated between my doctor(s) at Hartford Hospital and the University of Connecticut Medical Center. My thyroid condition has changed my life forever. I take my daily medication, have ongoing bouts of fatigue, voice fatigue due to one of my vocal cords being damaged from the surgery and a scar on my neck that people still ask me about. Overall, my medical journey was challenging and life-changing. However, I am so very grateful for the medical care and knowledge of the medical personnel and doctors who cared for me and who still do to this day.

## Consent

Written informed consent was obtained from the patient for publication of this case report and any accompanying images. A copy of the written consent is available for review by the Editor-in-Chief of this journal.

## Abbreviations

AITD: Autoimmune thyroid disease; FNAB: Fine-needle aspiration biopsy; IgM: Immunoglobulin M; IL: Interleukin; RA: Rheumatoid arthritis; RAI: Radioactive iodine; RF: Rheumatoid factor; SNP: Single nucleotide polymorphism; TNF-α: Tumor necrosis factor alpha; TSH: Thyroid-stimulating hormone; T3: Triiodothyronine; T4: Thyroxine.

## Competing interests

The authors declare that they have no competing interests.

## Authors’ contributions

AB contributed to conception and design, carried out the literature research, manuscript preparation and manuscript review. PH carried out the literature research, assisted with the manuscript preparation and manuscript review, and provided the histopathology. ST carried out the literature research, assisted with manuscript preparation and manuscript review. SV carried out the literature research, assisted with manuscript preparation and manuscript review. FF assisted with providing the histopathology data, as well as the preparation and interpretation of the biopsy and tissue specimens. BT contributed to conception and design, carried out the literature research, manuscript preparation and manuscript review. All authors read and approved the final manuscript.
